# Mcl1 as a Molecular Switch Linking Inflammatory Bowel Diseases to Colorectal Tumorigenesis

**DOI:** 10.3390/biom16050694

**Published:** 2026-05-07

**Authors:** Ahmed M. Elshazly, Jiong Li, Guang-Yu Yang, Senthil K. Radhakrishnan

**Affiliations:** 1 Department of Pathology, Virginia Commonwealth University, Richmond, VA 23298, USA; elshazlyam@vcu.edu (A.M.E.); guangyu.yang@vcuhealth.org (G.-Y.Y.); 2 Department of Pharmacology and Toxicology, Virginia Commonwealth University, Richmond, VA 23298, USA; 3 Department of Pharmacology and Toxicology, Faculty of Pharmacy, Kafrelsheikh University, Kafrelsheikh 33516, Egypt; 4 Department of Medicinal Chemistry, School of Pharmacy, Virginia Commonwealth University, Richmond, VA 23298, USA; jli29@vcu.edu; 5 Massey Comprehensive Cancer Center, Virginia Commonwealth University, Richmond, VA 23298, USA

**Keywords:** Mcl1, inflammatory bowel disease, colon cancer, therapy resistance

## Abstract

The maintenance of gastrointestinal homeostasis relies on a tightly coordinated interplay between the intestinal epithelium, the immune system, and the commensal microbiome. Disruption of this balance underlies inflammatory bowel disease (IBD), encompassing Crohn’s disease and ulcerative colitis, and is characterized by chronic, relapsing mucosal inflammation driven by genetic susceptibility, environmental factors, immune dysregulation, and microbial imbalance. Persistent inflammation promotes repeated cycles of epithelial injury and aberrant repair, creating a permissive environment for dysplasia and the development of colitis-associated cancer, most notably colorectal carcinoma. Recent evidence identifies the anti-apoptotic regulator myeloid cell leukemia-1 (Mcl1) as a critical determinant of epithelial integrity and cellular turnover during mucosal stress. Loss or destabilization of Mcl1 disrupts epithelial homeostasis, amplifies inflammatory signaling, and accelerates tumor initiation, whereas its adaptive upregulation in established malignancy promotes tumor cell survival, metabolic fitness, and therapeutic resistance. Thus, Mcl1 functions as a context-dependent molecular switch via restraining malignant transformation during chronic inflammation while supporting tumor progression once neoplasia is established. This functional duality positions Mcl1 as both a biomarker of disease progression and a therapeutically actionable vulnerability. In this review, we synthesize recent advances elucidating how Mcl1 integrates epithelial cell-fate decisions, immune signaling and tumor evolution across the IBD–cancer continuum. We further support these concepts through integrative analyses of multiple transcriptomic datasets comparing normal colonic mucosa with colorectal tumors, and we discuss emerging pharmacological strategies targeting Mcl1 in colitis-associated cancer.

## 1. Introduction

Inflammatory bowel diseases (IBD) are chronic, relapsing inflammatory disorders that primarily affect the gastrointestinal tract and are characterized by alternating periods of active inflammation and remission. The two major forms of IBD are Crohn’s disease (CD) and ulcerative colitis (UC). Clinical manifestations are heterogeneous and commonly include diarrhea, abdominal pain, rectal bleeding, and vomiting. Despite extensive investigation, the precise etiology of IBD remains incompletely understood. Current evidence suggests that disease pathogenesis arises from a complex interplay between intestinal microbiota dysbiosis, dysregulated immune responses, and genetic susceptibility [1]. Therapeutic management of IBD has evolved substantially and currently includes several drug classes, such as immunomodulators (e.g., thiopurines and methotrexate), biologic therapies (including anti-tumor necrosis factor agents, anti-integrin antibodies, and anti- interleukin(IL)-12/23 inhibitors), and, more recently, small-molecule therapies, such as Janus kinase (JAK) inhibitors and sphingosine-1-phosphate (S1P) receptor modulators [2,3]. Despite the clinical efficacy of these therapeutic agents, a substantial subset of patients remains at significantly increased risk of developing malignancies, particularly colorectal cancer, with cumulative incidence estimates ranging from 9 to 16% [3]. Currently, the standard of care for colon cancer arising in the context of IBD includes radiation [4], 5-fluorouracil/leucovorin combined with either oxaliplatin (FOLFOX) or irinotecan (FOLFIRI), together with a monoclonal antibody targeting VEGF (Vascular Endothelial Growth Factor) or EGFR (Epidermal Growth Factor Receptor), including bevacizumab, cetuximab, or panitumumab. Capecitabine-based regimens may also be employed as oral alternatives [5]. Notably, myeloid cell leukemia-1 (Mcl1) has emerged as a central mediator of therapeutic resistance to many of these agents across multiple malignancies, including resistance to radiation (Liang et al. [6]), 5-fluorouracil (Shi et al. [7]), oxaliplatin (Hu et al. [8], Fu et al. [9]), and irinotecan (Lin et al. [10]). Furthermore, recent studies have implicated Mcl1 in the transition from chronic intestinal inflammation to colorectal tumorigenesis, wherein Mcl1 exerts a protective role in the intestinal epithelium during inflammation, yet subsequently supports tumor cell survival and disease progression once malignant transformation has occurred. In this review, we highlight, for the first time to our knowledge, the dual and context-dependent roles of Mcl1 in gut epithelial homeostasis, inflammation-driven tumor initiation, and colorectal cancer progression, and summarize current advances in therapeutically targeting Mcl1 in colorectal cancer.

## 2. Mcl1 Overview

**Figure 1:**
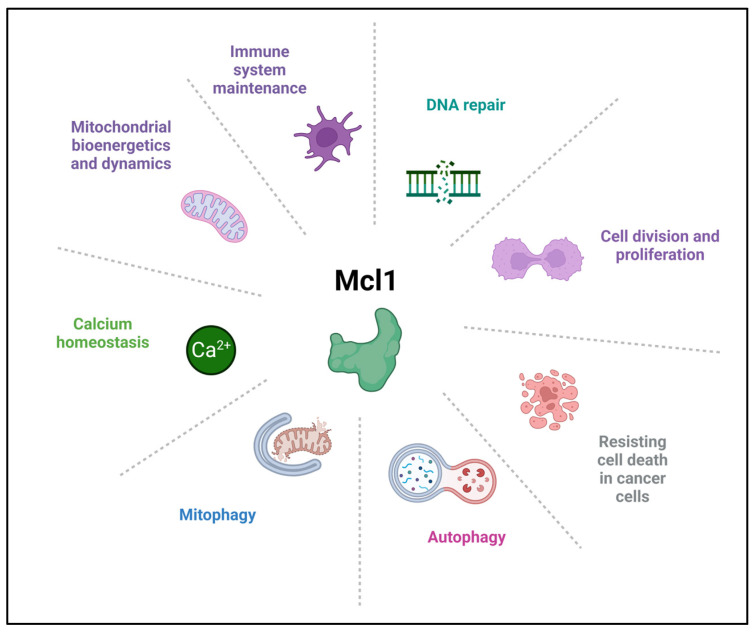
Mcl1 functions. Mcl1 has several cellular roles including cell survival, differentiation, cell division, DNA repair, immune system maintenance, autophagy, mitophagy, calcium homeostasis, mitochondrial bioenergetics and dynamics, in addition to the major antiapoptotic function.

Myeloid cell leukemia-1 (Mcl1) is a multifunctional cellular regulatory protein, best known for its anti-apoptotic role within the BCL-2 family. It was first identified by Kozopas and colleagues in 1993 in the laboratory of Ruth Craig, during efforts to identify genes associated with the transition from proliferation to differentiation in myeloid leukemia (ML-1) cells [11,12]. Mcl1 has been identified to play several cellular roles, including lineage-specific cell survival, differentiation, and maintenance, cell division, DNA repair, and immune system maintenance [13], autophagy and mitophagy, calcium homeostasis, mitochondrial bioenergetics and dynamics, in addition to the major antiapoptotic function (discussed by Widden and Placzek [11]) (Figure 1). The Mcl1 gene, located on chromosome 1p21.2, comprises three exons and encodes a 350-amino acid pro-survival protein, commonly referred to as Mcl1 (Mcl1 L). Alternative splicing of the Mcl1 transcript gives rise to two additional shorter isoforms, Mcl1S and Mcl1ES, which exert pro-apoptotic functions. Mcl1S behaves as a BH3-only-like sensor protein, selectively heterodimerizes with Mcl1L, promotes apoptosis, and displays a predominantly cytosolic localization. In contrast, Mcl1ES interacts with Mcl1L, localizes to the outer mitochondrial membrane (OMM), and induces BAK (BCL2 antagonist/killer)- and BAX (BCL2-associated X protein) -independent mitochondrial cell death. Additionally, the anti-apoptotic protein Mcl1 undergoes N-terminal proteolytic processing (cleavage) by the TOM/TIM import machinery and is translocated into the mitochondrial matrix, where it plays a critical role in maintaining mitochondrial integrity and regulating bioenergetic homeostasis [14,15,16,17,18,19].

The BCL-2 family of proteins is classified into three functional subgroups: anti-apoptotic members (including Mcl1, BCL-2 (B-cell lymphoma 2), BCL-XL, BCL-W, and BFL-1/A1), pore-forming effector proteins (BAX, BAK, and BOK), and pro-apoptotic BH3-only proteins (such as NOXA, BIM, and PUMA) [20]. All BCL-2 family proteins engage in dynamic protein–protein interactions to regulate the intrinsic apoptotic pathway by maintaining the integrity of the outer mitochondrial membrane (OMM). Upon irreparable cellular stress, upregulation of pro-apoptotic BH3-only proteins and downregulation or functional inactivation of pro-survival BCL-2 family members initiate apoptotic signaling. This process is accompanied by a reorganization of the BCL-2 family interactome, resulting in the liberation of the pore-forming effector proteins. Once released, BAX and BAK undergo conformational activation and homo-oligomerization, leading to the formation of cytotoxic pores within the OMM. These BAX/BAK oligomers facilitate the release of apoptogenic factors, such as cytochrome c, into the cytosol, thereby triggering rapid activation of the caspase cascade and execution of intrinsic apoptosis [21,22]. Specifically, Mcl1 binds to BAK, BAX, and BOK, inhibiting their activation, while being antagonized by PUMA [23,24,25]. Mcl1 has been extensively studied as a major mediator of resistance to multiple antineoplastic therapies [26,27,28]. These preclinical findings have been translated into several clinical trials; however, to date, no Mcl1 inhibitor has received FDA approval, largely due to concerns regarding dose-limiting cardiotoxicity [29,30], AMG 397 (NCT03465540), AZD 5991 (NCT03218683), and AMG 176 (NCT03797261).

## 3. Mcl1 as a Switch Between IBD and Carcinogenesis

Recent evidence has positioned Mcl1 as a pivotal guardian of intestinal homeostasis and a critical barrier to tumor initiation within the gastrointestinal tract. In a seminal study by Healy et al. [31], the authors generated intestinal epithelial cell (IEC)-specific Mcl1 knockout mice, thereby revealing the profound consequences of Mcl1 loss on mucosal integrity and inflammatory regulation. Compared with littermate controls, Mcl1^ΔIEC^ mice rapidly developed severe diarrhea, impaired weight gain, and markedly increased mortality, underscoring the essential role of Mcl1 in maintaining epithelial viability. Endoscopic evaluation revealed pronounced thickening and whitening of the colonic wall, accompanied by discrete polypoid lesions suggestive of preneoplastic transformation. These macroscopic abnormalities were accompanied by robust inflammatory activation, evidenced by elevated calprotectin in stool and tissues [32], as well as significantly increased circulating levels of pro-inflammatory cytokines (TNF-α, IL-22, IL-23A, IL-17A, IL-17F, and IL-1β). Importantly, these pathological phenotypes were observed exclusively following bi-allelic Mcl1 deletion, whereas monoallelic loss was insufficient to induce disease, indicating a non-redundant, dosage-dependent requirement for Mcl1 in maintaining epithelial homeostasis. Histopathological analyses demonstrated striking architectural derangement across both the small intestine and colon, characterized by crypt hyperplasia, villous atrophy, and widespread IEC apoptosis (cleaved caspase-3 positivity) rather than necroptosis. In parallel, hyperproliferative Ki-67^+^ crypts emerged, indicative of a maladaptive regenerative response. The mucosal microenvironment was profoundly remodeled, featuring expansive lymphoid aggregates enriched in B and T cells, together with a dramatic influx of innate and adaptive immune populations. Importantly, these findings were recapitulated in a tamoxifen-inducible IEC-specific Mcl1 conditional knockout model, which reproduced the full inflammatory and degenerative phenotype, validating the cell-autonomous requirement for Mcl1 in sustaining intestinal barrier integrity. Crucially, Mcl1 deficiency precipitated spontaneous malignant progression. Longitudinal monitoring revealed that >80% of Mcl1^ΔIEC^ mice developed intestinal neoplasms within one year, arising in both the small intestine and colon. These tumors spanned a full histopathological continuum, ranging from low- and high-grade adenomas to invasive carcinomas, all of which remained uniformly Mcl1-deficient in origin, thereby confirming a direct, cell-autonomous tumor-suppressive function for Mcl1 in the intestinal epithelium. Importantly, these findings directly support a role for Mcl1 loss in the initiation of dysplasia and tumorigenesis in the context of chronic inflammation, rather than solely in maintaining epithelial integrity. Strikingly, synteny analysis revealed a statistically significant overlap in chromosomal gains and losses between Mcl1-deficient tumors and human colorectal cancers. This genomic convergence was reinforced by gene set enrichment analysis (GSEA), which uncovered robust enrichment for transcriptional programs characteristic of human colorectal cancer, as well as those observed in intestinal tumors arising from IEC-specific adenomatous polyposis coli (Apc) deletion, a canonical model of intestinal tumorigenesis [33].

Mechanistically, Mcl1 loss disrupted intestinal stem cell (ISC) fate decisions, most notably by impairing differentiation toward secretory lineages. This defect was attributed to the downregulation of Atoh1, a transcription factor indispensable for secretory cell commitment, thereby establishing a direct mechanistic link between Mcl1 and lineage-specifying transcriptional programs. In parallel, the accumulation of DNA damage, evidenced by increased γH2AX staining, indicated a failure to preserve genomic integrity, further priming the epithelium for malignant evolution. Moreover, tamoxifen-inducible IEC-specific Mcl1 knockout models exhibited pronounced activation of wound-healing transcriptional programs and robust upregulation of Wnt target genes, as revealed by bulk RNA-seq analysis. Functional interrogation of this axis demonstrated that hyperactivation of Wnt signaling is necessary for the hyperproliferative state associated with Mcl1 loss: co-deletion of a single Ctnnb1 (β-catenin) allele, which encodes β-catenin, a central regulator of cell–cell adhesion and canonical Wnt signaling [34], was sufficient to partially rescue crypt hyperproliferation. Furthermore, pharmacologic inhibition of Wnt signaling using WNT974, an inhibitor for porcupine, which is an endoplasmic reticulum-resident O-acyltransferase required for Wnt protein palmitoylation, thereby blocking Wnt secretion and downstream Frizzled-dependent signaling [35], achieved complete normalization of crypt architecture and proliferative indices in both the small intestine and colon. Finally, to establish a direct relationship between Mcl1 deficiency and Wnt pathway activation, single-molecule fluorescence in situ hybridization (smFISH) revealed markedly increased Wnt2b expression within the stromal compartments of damaged, Mcl1-deficient regions, compared with adjacent, histologically intact areas retaining Mcl1 expression. These results demonstrate that Mcl1 deficiency-mediated hyperactivation is driven, at least in part, by Wnt signaling.

Deng et al. [36] investigated the contribution of microRNAs (particularly miR-452-5p) to intestinal homeostasis and inflammatory pathology, uncovering a functional connection to Mcl1 regulation. Expression profiling by qRT-PCR revealed that miR-452-5p levels were significantly elevated in the colons of dextran sulfate sodium (DSS)-induced ulcerative colitis (UC) mice relative to healthy controls. Functional inhibition of miR-452-5p markedly alleviated disease severity in vivo, as evidenced by a reduced disease activity index, attenuation of colon shortening, and diminished levels of the pro-inflammatory cytokines TNF-α, IL-6, and IL-8, collectively indicating a protective effect against colitis progression. Mechanistically, starBase target prediction identified Mcl1 mRNA as a putative binding target of miR-452-5p. Consistent with this, DSS-induced UC mice exhibited significantly decreased Mcl1 expression, inversely correlating with elevated miR-452-5p levels. Inhibition of miR-452-5p using anti-miR-452-5p restored Mcl1 expression in intestinal epithelial cells (IECs), even under conditions of enforced miR-452-5p overexpression (Ad-miR-452-5p), compared with mock-infected controls. These findings were corroborated in vitro in IEC-6 cells, where transfection with anti-miR-452-5p increased Mcl1 expression, while miR-452-5p mimics suppressed it. Finally, a dual-luciferase reporter assay confirmed direct binding of miR-452-5p to the Mcl1 3′-UTR, establishing Mcl1 as a bona fide post-transcriptional target of miR-452-5p.

Recently, Zeng et al. [37] uncovered a critical functional link between PHLDA1 (Pleckstrin homology-like domain, family A, member 1) and Mcl1 in regulating intestinal epithelial cell (IEC) apoptosis in the context of inflammatory bowel disease (IBD). PHLDA1 is a multifunctional protein, often acting as a tumor suppressor by inducing apoptosis and inhibiting AKT signaling [38]. Analysis of human patient samples revealed that PHLDA1 expression was markedly reduced in colonic tissues from individuals with Crohn’s disease and ulcerative colitis, compared with healthy controls. Notably, PHLDA1 levels inversely correlated with disease severity, as patients with moderate-to-severe IBD exhibited significantly lower expression than those with mild disease. Functionally, loss of PHLDA1 resulted in enhanced IEC apoptosis, leading to epithelial barrier disruption and facilitating the translocation of commensal bacteria into the mucosa. This breach in barrier integrity profoundly exacerbated intestinal inflammation, as demonstrated in mice with IEC-specific deletion of PHLDA1 (PHLDA1^IEC−KO^), which developed significantly more severe colitis compared with control animals. Mechanistically, the authors demonstrated that PHLDA1 physically interacts with Mcl1, as confirmed by co-immunoprecipitation and GST pull-down assays. Consistent with this interaction, Mcl1 protein levels were markedly reduced in PHLDA1-deficient mice, while Mcl1 mRNA levels remained unchanged, indicating post-translational regulation. Under dextran sulfate sodium (DSS) challenge, Mcl1 protein levels were notably reduced via proteasomal degradation, an effect that was rescued by the proteasome inhibitor MG132. Importantly, shRNA-mediated knockdown of PHLDA1 further decreased Mcl1 protein abundance in both HIEC-6 and NCM460 cells, compared with controls. Further investigation revealed that PHLDA1 deficiency enhanced Mcl1 poly-ubiquitination under DSS treatment both in vitro and in vivo. Strikingly, PHLDA1 loss was shown to promote K48-linked poly-ubiquitination of Mcl1 through the E3 ubiquitin ligase Mule, thereby targeting Mcl1 for proteasome-dependent degradation. These findings establish PHLDA1 as a critical stabilizer of Mcl1, acting through modulation of the ubiquitin–proteasome system. Importantly, the pathological consequences of PHLDA1 loss were functionally reversible: AAV-mediated restoration of Mcl1 expression or AAV-mediated knockdown of Mule significantly attenuated experimental colitis in PHLDA1^IEC−KO^ mice. Together, these data strongly demonstrate that PHLDA1 functions as a critical regulator of Mcl1 stability in intestinal epithelial cells by modulating its post-translational turnover. Specifically, PHLDA1 interacts with Mcl1 and limits its ubiquitination by the E3 ligase Mule, thereby preventing proteasomal degradation. Loss of PHLDA1 leads to enhanced Mcl1 degradation, resulting in increased epithelial cell apoptosis, disruption of barrier integrity, and exacerbation of intestinal inflammation. These findings position PHLDA1 as an important upstream regulator linking epithelial cell survival to inflammatory responses in IBD.

Collectively, these findings strongly establish Mcl1 as an essential regulator of intestinal epithelial cell homeostasis, where it safeguards epithelial survival, preserves barrier integrity, and restrains inflammation under physiological and inflammatory conditions. It is critical to highlight Zeng et al.’s [37] findings that DSS-induced ulcerative colitis alone leads to a reduction in Mcl1 levels, which is further exacerbated by loss of PHLDA1. These findings provide independent validation of earlier work by Healy et al. [31], which established that Mcl1 is essential for preserving epithelial homeostasis and preventing inflammation-driven tumorigenic transformation using various mouse models. However, this protective function becomes context-dependent: once epithelial cells acquire malignant traits, Mcl1 is upregulated and co-opted to support tumor cell survival, conferring resistance to apoptosis and facilitating disease progression. Initial clinical evidence supporting this biphasic model was provided by Nijhuis et al. [39], who demonstrated that Mcl1 expression levels were selectively diminished in fibrotic (stricturing) Crohn’s disease patient samples, whereas Mcl1 expression in non-fibrotic Crohn’s disease remained comparable to that observed in healthy controls. Furthermore, Seco-Cervera et al. [40], who reported that both Mcl1 protein and mRNA levels were significantly elevated in intestinal samples obtained from patients with complicated Crohn’s disease, suggesting that an initial reduction in Mcl1 levels, followed by sustained Mcl1 upregulation, may contribute to disease severity and progression toward malignancy. This evidence was further corroborated by Demelash et al. [41], who demonstrated markedly elevated Mcl1 expression in colorectal cancer (CRC). Specifically, these investigators reported substantially higher Mcl1 protein levels across multiple CRC cell lines, including SW480, SW620, SW837, DLD-1, Caco2, RKO, HT-29, and HRT-18 cells, compared with primary colonic epithelial cells (CRL-1459). Consistent with these in vitro observations, analyses of CRC patient specimens revealed significantly increased Mcl1 expression at both the protein and mRNA levels relative to normal colonic epithelium, reinforcing the notion that Mcl1 upregulation is a recurrent feature of colorectal tumorigenesis. It is also critical to highlight Mittal et al.’s [42] results demonstrating the critical role of Mcl1 in the immune response to colorectal cancer. They reported that high Mcl1 expression in colorectal cancer samples was associated with higher mutation rates of BCOR, TP53, KMT2D, ASXL1, KDM6A, ATM, MSH6, SPEN, KRAS, STK11, GNAS, RNF43, in addition to the upregulation of PD-L1, B cells, natural killer cells, T-reg cells, and M1 and M2 macrophages infiltration. Thus, elevated Mcl1 expression identifies colorectal tumors with high mutational load and a profoundly altered immune microenvironment.

**Figure 2:**
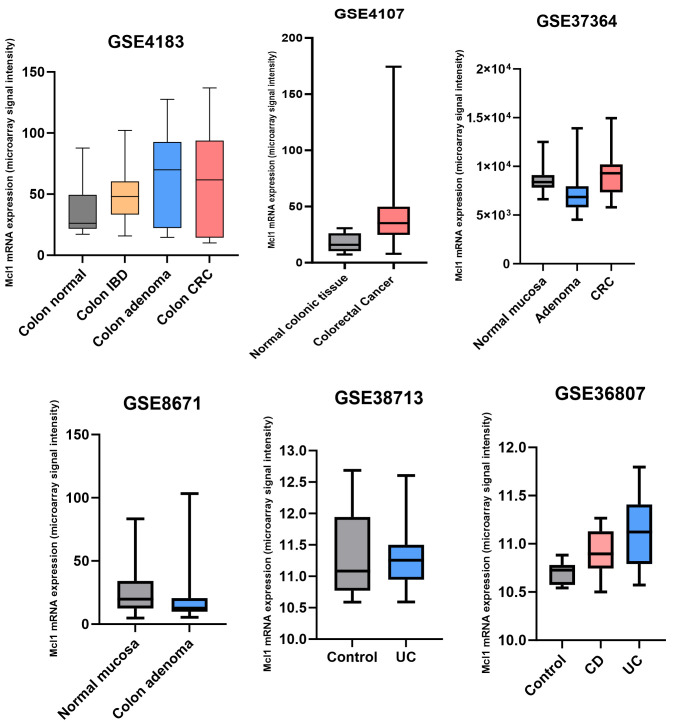
Dynamic regulation of Mcl1 expression across the inflammatory bowel disease–colorectal cancer continuum. Box plots depict Mcl1 mRNA expression across multiple independent transcriptomic datasets encompassing normal colonic mucosa, inflammatory bowel disease (IBD), adenomas, ulcerative colitis (UC), Crohn’s disease (CD) and colorectal cancer (CRC).

To further support our conclusions, we analyzed several publicly available transcriptomic datasets (GSE4183, GSE8671, GSE4107, GSE37364, GSE38713 and GSE36807) comprising normal colonic mucosa, adenomas, and colorectal cancer samples. As shown in Figure 2, Mcl1 expression exhibits a dynamic shift from low levels in normal mucosal tissues to increased expression in colorectal cancer across multiple datasets. Notably, GSE8671, GSE38713 and GSE37364 support a biphasic model in which Mcl1 expression is initially reduced at the precancerous stages before becoming upregulated in established carcinoma, a pattern that was not consistently observed in GSE4183 and GSE36807. Importantly, these transcript-level changes may not fully capture corresponding alterations at the protein level, underscoring the need for complementary proteomic and functional analyses.

## 4. Therapeutic Targeting of Mcl1 in IBD-Derived Cancers

Preclinical evidence strongly supports targeting Mcl1 in IBD-derived cancers as a promising therapeutic strategy, not only for the initial elimination of malignant cell populations but also for overcoming the emergence of therapeutic resistance. For example, Ulrich-Pur et al. [43] analyzed Mcl1 expression in 23 rectal cancer specimens. Half of the patients received preoperative short-course radiotherapy (25 Gy) followed by radical surgery, whereas the remaining patients underwent surgery alone. Notably, high Mcl1 expression was detected in 35% of all tumors, with markedly stronger expression observed in irradiated compared with non-irradiated specimens. These findings suggest that Mcl1 upregulation may limit the therapeutic efficacy of radiation therapy and contribute to treatment resistance in rectal cancer. Consistently, Yu et al. [44] reported that genetic depletion of Mcl1 markedly sensitized HCT116 and HT29 colorectal cancer cells to irradiation, leading to a pronounced reduction in cell viability and clonogenic survival, accompanied by increased caspase-3 activation. In contrast, Mcl1 overexpression attenuated radiation-induced cytotoxicity and preserved both cell viability and colony-forming capacity.

Tong et al. [45] demonstrated that Mcl1 is a central determinant of resistance to multiple kinase inhibitors, and that such resistance can be effectively abolished through either genetic or pharmacologic inhibition of Mcl1. Using colorectal cancer models, the authors first showed that several multi-kinase inhibitors, including the FDA-approved agents regorafenib, sorafenib, and sunitinib, induced rapid depletion of Mcl1 protein at apoptosis-inducing concentrations in HCT116 cells. This effect was consistently reproduced across a diverse panel of colorectal cancer cell lines, irrespective of KRAS, BRAF, PIK3CA, or TP53 mutational status, including RKO, DLD1, HT29, and Lim2405, underscoring the broad relevance of Mcl1 regulation in colorectal cancer. Importantly, Mcl1 depletion was completely prevented by the proteasome inhibitor MG132, establishing that kinase inhibitor-induced Mcl1 loss occurs via a ubiquitin–proteasome-dependent degradation mechanism rather than transcriptional repression.

Mechanistically, regorafenib was shown to inhibit CRAF-ERK signaling, thereby relieving inhibitory phosphorylation of GSK3β, which in turn promoted GSK3β-dependent phosphorylation of Mcl1. This phosphorylation event facilitated FBW7 binding, triggering poly-ubiquitination and proteasomal degradation of Mcl1. To directly test the requirement of Mcl1 phosphorylation for this process, the authors generated phospho-deficient Mcl1 mutants (S121A/E125A/S159A/T163A). Strikingly, HCT116 cells expressing these Mcl1 phosphorylation-site mutants exhibited near-complete abrogation of regorafenib-induced Mcl1 degradation, FBW7 association, and ubiquitination. Functionally, these mutant cells displayed profound resistance to regorafenib and sorafenib, characterized by enhanced cell viability, increased clonogenic survival, and marked suppression of apoptosis, as assessed by nuclear fragmentation, Annexin V/PI staining, and caspase-3, -8, and -9 activation. The causal dependence on Mcl1 was further confirmed by Mcl1 knockdown, which fully restored regorafenib sensitivity in mutant HCT116 cells. Consistently, treatment with the selective Mcl1 inhibitor UMI-77 completely reinstated regorafenib-induced apoptosis and caspase activation in the resistant mutant cells. These findings were further validated in vivo, where wild-type HCT116 xenografts exhibited significant tumor regression in response to regorafenib, whereas xenografts derived from cells expressing phosphorylation-resistant Mcl1 mutants were largely refractory to treatment. Overall, this study establishes Mcl1 degradation as a mechanistic linchpin of kinase inhibitor-induced apoptosis and identifies Mcl1 stabilization as a dominant driver of therapeutic resistance in colorectal cancer.

In a follow-up study from the same group, Song et al. [46] generated regorafenib-resistant HCT116 and LIM1215 cell lines and showed that co-treatment with regorafenib and the Mcl1 inhibitors S63845, AZD5991, or AMG176 restored regorafenib sensitivity to levels comparable to those observed in the parental HCT116 and LIM1215 cells. These in vitro observations were recapitulated in vivo, where pharmacologic inhibition of Mcl1 with S63845 reinstated regorafenib responsiveness in HCT116-resistant xenograft tumors.

Recently, Gan et al. [47] investigated the preclinical therapeutic potential of Mcl1 degradation in colorectal cancer using the natural product bergenin [48]. They initially demonstrated that bergenin significantly inhibited the proliferation of HCT116, HT29, and SW620 cells, as assessed by MTS and colony-formation assays. Consistent with induction of apoptosis, bergenin treatment markedly increased the levels of cleaved caspase-3 and cleaved PARP in HCT116 and HT29 cells. These anti-tumor effects were recapitulated in vivo, where bergenin significantly delayed tumor growth in HCT116- and HT29-derived xenograft models, accompanied by a substantial reduction in tumor weight. Mechanistically, the authors showed that bergenin exerts its antitumor activity through post-translational degradation of Mcl1. Bergenin markedly reduced Mcl1 protein levels without affecting Mcl1 mRNA expression in both HCT116 and HT29 cells, while co-treatment with the proteasome inhibitor MG132 restored Mcl1 protein abundance, indicating ubiquitin–proteasome system-dependent degradation. Further mechanistic analyses revealed that bergenin promotes K48-linked ubiquitination of Mcl1 and enhances its interaction with the E3 ubiquitin ligase FBW7, thereby facilitating proteasomal degradation. In addition, bergenin was shown to suppress AKT signaling, resulting in activation of GSK3β, a known upstream regulator of FBW7-mediated Mcl1 turnover [49]. Consistently, AKT1 overexpression attenuated the bergenin-induced reduction in Mcl1 protein levels, while genetic knockdown of GSK3β restored Mcl1 expression in bergenin-treated HCT116 cells, supporting a model in which bergenin induces FBW7-mediated Mcl1 degradation in an AKT-GSK3β signaling-dependent manner.

Notably, Mcl1 degradation via the FBW7 E3 ligase appears to represent a recurrent regulatory mechanism in colorectal cancer. Tong et al. [45] and Gan et al. [47] reported Mcl1 degradation in response to kinase inhibitor or bergenin through the FBW7 E3 ligase under the control of the AKT/GSK3β axis. This signaling framework was further reinforced by Yu et al. [44], who demonstrated that the E3 ligase Skp2 stabilizes Mcl1 and that elevated Skp2 expression in colorectal cancer correlates with increased Mcl1 levels. Importantly, genetic knockout or depletion of Skp2 enhanced FBW7-mediated Mcl1 ubiquitination, resulting in Mcl1 degradation and increased radiosensitivity. This evidence critically underscores the importance of the ubiquitinated–proteasomal system in Mcl1 stability and eventually its role in resistance development.

## 5. Discussion and Challenges

In this review, we synthesize current evidence to propose a model in which Mcl1 functions as a context-dependent regulator in IBD-associated tumorigenesis, with its role determined by both disease stage and cellular context. For clarity, this progression can be conceptualized across three stages: early inflammatory injury, chronic inflammation, and dysplasia-to-carcinoma transition. During early inflammatory stages, Mcl1 is preferentially destabilized in intestinal epithelial cells through coordinated post-transcriptional and post-translational mechanisms, including microRNA-mediated repression (e.g., miR-452-5p), which represents one of several mechanisms regulating Mcl1 expression, and enhanced ubiquitin–proteasome degradation mediated by E3 ligases such as Mule [36,37]. This reduction in Mcl1 compromises epithelial cell survival, disrupts barrier integrity, and promotes compensatory hyperproliferation through aberrant Wnt signaling within a pro-inflammatory microenvironment, thereby facilitating early tumorigenic events [31]. Loss of Mcl1 in intestinal epithelial cells also leads to increased exposure to luminal antigens, triggering activation of immune responses [31,50]. Mcl1 is essential for the survival of multiple immune cell populations, including T cells, B cells, and neutrophils, and its dysregulation may influence inflammatory signaling and immune cell persistence [13,51,52,53]. This interplay between epithelial damage and immune activation contributes to the establishment of a pro-inflammatory microenvironment that promotes disease progression and chronic inflammation.

**Figure 3:**
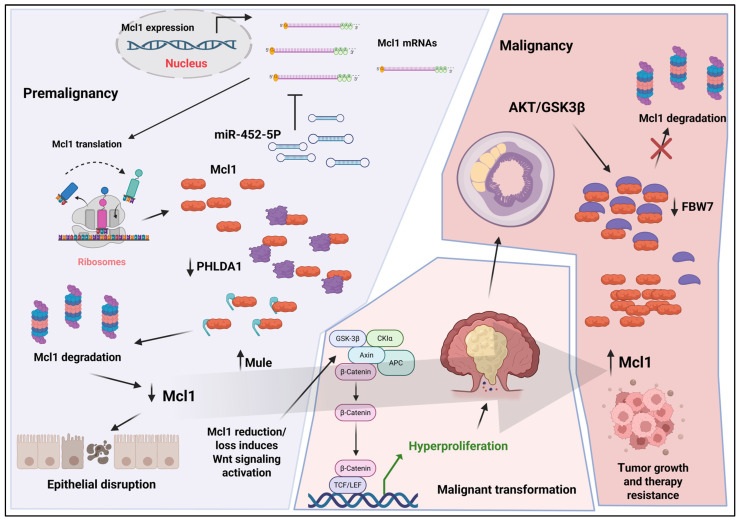
Mcl1 functions as a molecular switch governing the transition from inflammatory bowel disease to colorectal cancer. During early inflammatory (pre-malignant) stages, Mcl1 expression is reduced in intestinal epithelial cells through coordinated post-transcriptional and post-translational mechanisms, including microRNA-mediated repression (e.g., miR-452-5p) and enhanced ubiquitination by the E3 ligase Mule following PHLDA1 loss, leading to proteasomal degradation. This reduction compromises epithelial cell survival, disrupts barrier integrity, and promotes compensatory hyperproliferation through aberrant activation of Wnt signaling. As inflammation becomes chronic, a tumor-promoting microenvironment characterized by immune cell activation and sustained signaling facilitates the accumulation of oncogenic alterations. In established colorectal cancer, oncogenic pathways, including the AKT/GSK3β axis, promote Mcl1 stabilization by limiting FBW7-mediated degradation, resulting in Mcl1 accumulation. Elevated Mcl1 suppresses mitochondrial apoptosis through sequestration of BAX and BAK, thereby promoting tumor cell survival, metabolic adaptation, and resistance to therapy. Together, these events drive progression from chronic inflammatory bowel disease to tumor initiation and colorectal cancer.

As inflammation becomes chronic, Mcl1 regulation appears more heterogeneous across epithelial and immune cell populations, although the precise mechanisms governing this intermediate state remain incompletely defined. In later stages, particularly during dysplasia and established colorectal cancer, oncogenic signaling pathways, including modulation of the AKT/GSK3β axis, promote Mcl1 stabilization by limiting FBW7-dependent ubiquitination and degradation, resulting in its accumulation [45,46,47]. In tumor cells, elevated Mcl1 suppresses mitochondrial apoptosis through sequestration of BAX and BAK, thereby promoting cell survival, metabolic adaptation, and resistance to therapy. Importantly, this model reframes the “molecular switch” not as a binary transition but as a dynamic equilibrium governed by opposing regulatory inputs controlling Mcl1 turnover and function in a stage- and cell-type-specific manner. This framework provides a mechanistic basis for the dual role of Mcl1 across the IBD–colorectal cancer continuum and supports the need for context-specific therapeutic strategies targeting Mcl1 regulation (Figure 3).

Accumulating evidence from preclinical models and transcriptomic analyses consistently demonstrates elevated Mcl1 expression in colorectal cancer, with profound implications for genomic instability and immune cell infiltration within the tumor microenvironment. This evidence further underscores the critical role of Mcl1 in the development of resistance to multiple standard-of-care therapies, thereby limiting their clinical efficacy.

Despite its therapeutic appeal, the clinical development of Mcl1 inhibitors remains constrained across tumor types, including colorectal cancer. Beyond its canonical anti-apoptotic function [11,15,27,54] (Table 1). Mcl1 is essential for hematopoietic stem cells [55] and, more critically, for maintaining cardiac integrity [17]. Cardiomyocyte-specific deletion of Mcl1 results in rapidly progressive dilated cardiomyopathy, mitochondrial disorganization, and impaired oxidative respiration [56]. Moreover, Mcl1-deficient hearts exhibit mitochondrial swelling, permeability transition pore opening, myocyte rupture, and accumulation of dysfunctional mitochondria [17,18]. Mcl1 is also required for PINK1-Parkin-mediated mitophagy and proper autophagic flux, processes that are critical for mitochondrial quality control and cardiomyocyte viability [17,18,57]. Consistent with these essential cardiac roles, several clinical trials of Mcl1 inhibitors have been discontinued due to cardiotoxicity and troponin elevation, including AMG-397 (NCT03465540), AZD5991 (NCT03218683), and AMG-176 (NCT03797261) [29,30]. At present, PRT1419 remains in clinical trials, and no Mcl1-targeting agent has yet demonstrated a consistently safe cardiac profile [58]. These limitations highlight a major barrier to clinical translation and underscore the need for future strategies aimed at improving the therapeutic window of Mcl1 targeting.

As discussed throughout this review, resistance mechanisms frequently converge on pathological accumulation of Mcl1 through inhibition of its degradation. Accordingly, emerging therapeutic strategies aim to promote Mcl1 elimination [59], either via proteasomal degradation [60] or autophagy-mediated turnover [61]. Notably, leveraging autophagy to degrade Mcl1 may be particularly advantageous in colorectal cancer, where autophagy often serves a cytoprotective and therapy-adaptive function [62,63,64]. Collectively, these findings establish Mcl1 as a context-dependent molecular switch that is indispensable for epithelial integrity during inflammatory stress, yet co-opted in cancer to drive therapeutic resistance and tumor persistence.

## 6. Conclusions

In summary, mounting evidence positions Mcl1 as a central, context-dependent regulator across the inflammatory bowel disease–colorectal cancer continuum. By integrating signals that govern epithelial survival, immune responses, and tumor cell fitness, Mcl1 functions as a dynamic molecular switch that both restrains early inflammation-driven tumor initiation and, paradoxically, supports tumor progression and therapeutic resistance once malignancy is established. This duality highlights the complexity of targeting Mcl1 therapeutically. While inhibition of Mcl1 represents a promising strategy to overcome resistance in colorectal cancer, its essential roles in normal tissue homeostasis, particularly in the heart and immune system, pose significant challenges for clinical translation. Moving forward, a deeper understanding of the regulatory mechanisms controlling Mcl1 expression and stability, as well as the development of context-specific or tissue-selective targeting approaches, will be critical. Ultimately, leveraging the stage-specific functions of Mcl1 may enable more precise therapeutic interventions, improving outcomes for patients with IBD-associated colorectal cancer while minimizing adverse effects.

## Figures and Tables

**Table 1:** Recent advances in preclinical and clinical targeting of Mcl1 in colon or solid malignancies.

Model/System	Mcl1 Targeting Strategy	Treatment Context	Key Outcome	Stage	Reference
CRC cell lines (HCT116, HT29)	Genetic depletion (knockdown)	Radiation therapy	Mcl1 depletion significantly sensitized cells to irradiation	Preclinical	[44]
CRC cell lines + xenografts	Genetic knockdown + pharmacologic inhibition (UMI-77)	Kinase inhibitors (regorafenib, sorafenib)	Mcl1 targeting restored drug sensitivity and apoptosis	Preclinical	[45]
Resistant CRC cell lines + xenografts	Pharmacologic inhibition (S63845, AZD5991, AMG176)	Regorafenib-resistant models	Combination therapy overcame acquired resistance	Preclinical	[46]
CRC cell lines + xenografts	Indirect pharmacologic degradation (bergenin)	NA	Induced apoptosis and suppressed tumor growth	Preclinical	[47]
Advanced/metastatic solid tumors	PRT1419	NA	Phase 1 trial showed acceptable safety with no cardiac toxicity, and stable disease was achieved in some patients.	Clinical	NCT04837677

## Data Availability

A systematic search of the Gene Expression Omnibus (GEO) database (https://www.ncbi.nlm.nih.gov/geo/ (accessed on 2 January 2026)) was performed to identify publicly available transcriptomic datasets containing MCL1 expression across relevant pathological conditions, including normal colonic mucosa, inflammatory bowel disease (IBD), adenomas, ulcerative colitis (UC), Crohn’s disease (CD), and colorectal cancer (CRC). Datasets were selected based on the availability of expression data and clearly annotated clinical groups. Expression data were explored using GEO2R, an interactive web-based tool that allows visualization and comparison of gene expression across predefined sample groups. No additional statistical analyses or cross-dataset normalization were performed beyond the default processing provided by GEO2R. Mcl1 expression values were extracted from each dataset and plotted using GraphPad Prism 10 to enable visual comparison of expression trends across disease states. This analysis was intended to provide a qualitative, exploratory overview of Mcl1 expression patterns rather than a formal meta-analysis.
